# Suitability of internal transcribed spacers (ITS) as markers for the population genetic structure of *Blastocystis* spp

**DOI:** 10.1186/s13071-014-0461-2

**Published:** 2014-10-03

**Authors:** Guiehdani Villalobos, Guadalupe Erendira Orozco-Mosqueda, Merle Lopez-Perez, Eduardo Lopez-Escamilla, Alex Córdoba-Aguilar, Lucia Rangel-Gamboa, Angelica Olivo-Diaz, Mirza Romero-Valdovinos, Pablo Maravilla, Fernando Martinez-Hernandez

**Affiliations:** Departamento de Ecología Evolutiva, Instituto de Ecología, Universidad Nacional Autónoma de México, Apdo. Postal 70-275, Ciudad Universitaria, 04510 Mexico, DF Mexico; Hospital Infantil de Morelia “Eva Samano de Lopez Mateos”, Bosque Cuauhtemoc s/n, Morelia, 58000 Michoacan Mexico; Hospital General “Dr. Manuel Gea Gonzalez”, Calzada de Tlalpan 4800, Mexico, 14080 DF Mexico

**Keywords:** *Blastocystis spp*, Internal transcribed spacers, *Blastocystis* subtypes, Genetic variation

## Abstract

**Background:**

The purpose of this study was to assess the genetic variation and differentiation of *Blastocystis* subtypes (STs) recovered from symptomatic children by analysing partial sequences of the small subunit rDNA gene region (SSUrDNA) and internal transcribed spacers (1 and 2) plus the 5.8S region (ITS, ITS1 + 5.8S + ITS2) and comparing with isolates from other countries.

**Findings:**

Faecal samples from 47 *Blastocystis*-infected children with gastrointestinal symptoms and negative for pathogenic enterobacteria were analysed. PCR was performed on DNA from all the samples to identify *Blastocystis* STs, amplifying a fragment of SSUrDNA and the ITS region. The amplicons were purified and sequenced, and consensus sequences were submitted to GenBank; afterwards, SSUrDNA sequences were analysed for genetic diversity according to geographic area. Regarding the *Blastocystis* STs found, 51% were ST1, 23% ST2, 19% ST3 and 2% ST7. For ITS, a haplotype network tree and Bayesian inference revealed the presence of two novel variants of ST1, clustering some sequences into ST1A and ST1B. The values of nucleotide diversity (π) and haplotype polymorphism (θ) for ST1, ST2 and ST3 ranged from 0 to 1, whereas the ratio of genetic differentiation (F_ST_)/migration index (Nm) showed the highest differentiation between Libya and Thailand-Philippines for ST2 (0.282/0.63). In contrast, a high flow gene was observed between Czech Republic-Denmark-Holland-Spain and USA-Mexico-Colombia for ST1 (0.003/84).

**Conclusion:**

Our data on genetic differentiation and gene flow might explain the differences for the prevalence of *Blastocystis* STs. Moreover, the ITS region could be used as a genetic marker to assess genetic variation in this parasite.

## Findings

*Blastocystis* spp are very common intestinal parasites of humans and animals, with a prevalence that varies from country to country and among various communities within the same country [1-3]. In addition, this parasite shows extensive polymorphisms, as reflected by microscopy or molecular analysis. Such polymorphisms have allowed for the recognition of at least 17 genetic subtypes (STs) in the genus *Blastocystis* based on the analysis of its small subunit rDNA (SSUrDNA) [2,4-8]. In eukaryotes, DNA sequences coding for rDNA genes constitute transcription units located on nuclear chromosomes, which are organised to form a simple multigenic family comprising a high number of repeated copies in tandem. The internal transcribed spacer includes two spacers, ITS1 and ITS2, separated by the 5.8S rRNA gene. These spacer regions evolve much faster than coding regions because substitutions occurring in spacers may be considered neutral mutations without any constraints. Therefore, the large amount of research available with regard to the usefulness of ITS sequences as excellent markers for species distinction has led scientists to consider both, but mainly ITS-2, as the best tool for problematic taxa, such as cryptic species. Furthermore, the 5.8S rRNA gene, although being too short to effectively indicate robust phylogenies across large time scales, shows levels of gene conservation similar to SSUrDNA, which is the most frequently used region for genetic typing analyses in *Blastocystis* [9,10].

As there is scarce knowledge of the evolution, ecology, and population genetics in this parasite, the purpose of the present study was to assess the genetic variation of *Blastocystis* STs recovered from symptomatic children from Michoacan state, Mexico, by analysing partial SSUrDNA sequences and internal transcribed spacers (1 and 2) plus the 5.8S region (ITS, ITS1 + 5.8S + ITS2) and comparing with isolates from other countries.

Frozen faecal samples from 47 children infected by *Blastocystis* (22 males, 25 females, mean age of 8 ± 3.3 years), who were treated at the Hospital Infantil de Morelia “Eva Samano de Lopez Mateos” due to gastrointestinal symptoms and were negative for pathogenic enterobacteria, were analysed. Clinical and demographic data were obtained from medical records, and information about the *Blastocystis* load (reported as “more than” or “less than” five *Blastocystis* cells per 40X field) was obtained from laboratory files; variables were analysed by χ^2^ tests. The Ethics and Research Committee of the hospital approved the study, and informed consent was obtained from one parent of each child.

Stool DNA was extracted from approximately 250 mg faeces using QIAamp DNA Stool Mini Kit (QIAGEN Inc., Germany). A partial SSUrDNA sequence (~500 bp) was obtained using the primers reported by Santín *et al.* [11] A new specific set of primers for amplifying the ITS1 + 5.8S + ITS2 (ITS) region was designed based on the alignment of highly conserved areas that delimited this region of available *Blastocystis* sequences and other related organisms in GenBank (*Blastocystis hominis*, AY125914-AY125919; *Proteromonas lacertae*, AY224080; *Fucus vesiculosus,* EF625890, AF102930, AF102913, AF102920, AF102929; *Thalassiosira pseudonana,* HF565129-HF565132; *Laminaria digitata,* FJ042772, FJ042773, FJ042764). The designed forward and reverse primers were ITS_Blas_F (5′-GGAAGGTGAAGTCGTAACAAG-3′) and ITS_Blas_R (5′-CAGCAGGTCTTCTTRCTTGA-3′).

DNA (2 μL) was used to amplify the genomic sequences in a 25-μL PCR reaction. For ITS1-5.8S-ITS2 region amplification, the reaction contained 200 pmol of each nucleotide, 1X PCR buffer (8 mM Tris–HCl, pH 8, 20 mM KCl, 1 mM MgCl_2_), 1X dNTPs, 0.01 mg BSA, and 1 U *Taq DNA Polymerase* (Promega). After the first denaturation step at 94°C for 5 min, 35 cycles of denaturation at 94°C for 30 s, annealing at 60°C for 45 s, and extension at 72°C for 30 s were performed, followed by a final extension step at 72°C for 10 min. The amplicons were subjected to electrophoresis in 1.2% agarose gels and then purified and sequenced on both strands by a commercial service.

All sequences were subjected to a BLAST search in the GenBank database; multiple alignments were performed using the CLUSTAL W [12] and Muscle [13] programmes with manual adjustment in MEGA 5.05 software [14]. The best fit model of nucleotide substitution was determined using the Akaike Information Criterion in Modeltest version 3.7 software [15]; for both markers, it was the Hasegawa Kishino Yano model with gamma distribution and invariable sites. The phylogenetic reconstruction using Bayesian inference was performed with the Mr. Bayes 3.1.2 programme [16-18]. The analysis was performed for 10 million generations with sampling trees every 100 generations. Trees with scores lower than those at the stationary phase (burn-in) were discarded, and the trees that reached the stationary phase were collected and used to build majority consensus trees. Other sequences of both markers were obtained from GenBank and used as subtype references. A Median Joining Network analysis [19] was generated using NETWORK 4.611 software (fluxus-engineering.com) with the default settings and assumptions. A genetic diversity analysis within and between populations was performed using DnaSPv4 [20] and included nucleotide diversity (π), haplotype polymorphism (θ), gene flow (Nm) and genetic differentiation index (F_ST_). These indexes refer to the following: π, the average proportion of nucleotide differences between all possible pairs of sequences in the sample; θ, the proportion of nucleotide sites that are expected to be polymorphic in any suitable sample from this region of the genome. Both indexes are used to assess polymorphisms at the DNA level and to monitor diversity within or between ecological populations and examine the genetic variation in related species or their evolutionary relationships. F_ST_ is a typical genetic statistic used to measure differentiation between or among populations. The common used values for genetic differentiation are as follows: 0 to 0.05, small; 0.05 to 0.15, moderate; 0.15 to 0.25, great; values above 0.25 indicate huge genetic differentiation. The gene flow or migration index (Nm) refers to the movement of organisms among subpopulations; those strongly differentiated have an Nm < < 1, whereas an Nm > 4 behaves as a single panmictic unit [21].

Table 1 summarises the clinical, parasitological and genetic data of the infected children. Although 47 faecal samples were analysed, clinical information for 7 patients was not recovered because the clinical records were incomplete and the parent who gave informed consent was not sure of the symptoms. Co-infection was common with other commensal single protozoa including *Endolimax nana* (19%), *Entamoeba coli* (15%) and *Entamoeba hartmanni* (13%). Only 10 samples (21%) presented >5 *Blastocystis* stages per 40X field, with the vacuolar form being the most frequent stage found. Regarding *Blastocystis* STs, 43 sequences were obtained for SSUrDNA and 46 for ITS. For five samples, we did not have a sufficient amount of DNA with adequate quality to obtain amplicons, most likely due to the activity of several DNases that degrade nucleic acids and therefore decreased the DNA concentration in the samples [22]. A BLAST search confirmed that each sequence matched *Blastocystis*. All amplicons for SSUrDNA were ~500 bp. For ITS, the amplicons ranged from 530 to 620 bp, showing products of ~550, ~530, ~620 and ~590 bp for ST1, 2, 3 and 7, respectively. Regarding *Blastocystis* STs, 51% were ST1, 23% ST2, 19% ST3 and 2% ST7 by SSUrDNA and/or ITS. Only two cases (samples 17 and 34) did not match, which might have been due to a phenomenon of co-infection between STs because the sequencing of several clones is required to detect potential co-infections by different STs when SSUrDNA markers are used. No statistical associations between the symptoms, parasite load and *Blastocystis* STs were found.

**Table 1:** **Demographic, clinical data and** ***Blastocystis*** **subtype in children from Michoacan, Mexico**

**Sample**	**Gender/age**	**Symptoms**	**Co-infections**	Parasite load*	**Subtype (GenBank access)**	
					**SSUrDNA** ^**‡**^	**ITS** ^**§**^
1	M/8	6,7,10	-	<5	1 (KM213425)	1B (KM213469
2	F/9	1,4,7,10	*Endolimax nana Entamoeba histolytica/E. dispar*	<5	1 (KM213426)	ND
3	M/5	ND	-	>5	1 (KM213427)	1A (KM213470)
4	F/10	2,3,4,5,7,8,9,10	*E. nana*, *Entamoeba hartmanni*	>5	1 (KM213428)	1B (KM213471)
5	F/7	1,2,3,4,6,9,10	-	<5	1 (KM213429)	1B (KM213472)
6	F/5	ND	*Entamoeba coli*	>5	1 (KM213430)	1B (KM213473)
7	M/9	1,2,3,4,5,6,10,11	-	>5	1 (KM213431)	1B (KM213474)
8	M/4	ND	-	<5	ND	1A (KM213475)
9	M/13	3,4,6,7,9,11	-	<5	1 (KM213432)	1B (KM213476)
10	M/9	1,2,3,4,5,7,8,9,11	*E. nana*, *E. coli*	>5	1 (KM213433)	1A (KM213477)
11	M/4	1,3,6,7	*E. coli*	<5	1 (KM213434)	1B (KM213478)
12	F/10	2,3,4,8,9,10	-	>5	1 (KM213435)	1B (KM213479)
13	M/9	1,2,3,4,7	-	>5	1 (KM213436)	1A (KM213480)
14	F/5	2,3,6,10	*E. nana*	<5	1 (KM213437)	1B (KM213481)
15	M/13	4,10,11	*E. coli*	<5	1 (KM213438)	1B (KM213482)
16	F/5	4,10,11	*E. nana*	<5	1 (KM213439)	1B (KM213483)
17	F/7	2,10	-	<5	1 (KM213440)	3 (KM213484)
18	F/5	4,6,10	-	<5	1 (KM213441)	1A (KM213485)
19	F/7	4,7	*E. nana*	<5	1 (KM213442)	1A (KM213486)
20	M/7	4,7	-	<5	1 (KM213443)	1A (KM213487)
21	F/4	2,4,6,10	*E. nana*	<5	1 (KM213444)	1B (KM213488)
22	F/5	2,3,5,6,7,8,11	-	<5	1 (KM213445)	ND
23	M/10	4,6,7	-	<5	1 (KM213446)	1B (KM213489)
24	M/7	5,6	*E. nana*	<5	1 (KM213447)	1A (KM213490)
25	F/14	1,2,3,4,7,8,9,10	-	<5	1 (KM213448)	1B (KM213491)
26	F/5	1,2,3,4,7	-	<5	2 (KM213449)	2 (KM213492)
27	M/4	3,4,6,7	-	<5	2 (KM213450)	2 (KM213493)
28	F/9	2,3,8	*E. histolytica/E. dispar*, *E. nana*	<5	2 (KM213451)	2 (KM213494)
29	M/11	1,4,7	*E. coli*	>5	2 (KM213452)	2 (KM213495)
30	F/6	1,2,3,6,7,10,11	*E. coli*, *E. histolytica/E. dispar, E. hartmanni*	<5	2 (KM213453)	2 (KM213496)
31	F/6	ND	*E. coli*, *E. hartmanni*	<5	ND	2 (KM213497)
32	F/7	1,2,3,4,7	-	<5	ND	2 (KM213498)
33	M/13	ND	Microsporidia	<5	2 (KM213454)	2 (KM213499)
34	M/1	1,2,4,5,8,10	*E. hartmanni*	<5	2 (KM213455)	1B (KM213500)
35	F/7	2,3,5,7,9	*E. coli*	>5	2 (KM213456)	2 (KM213501)
36	M/13	8,10	-	<5	2 (KM213457)	2 (KM213502)
37	F/10	2,3,7,8,9,10	*E. hartmanni*	<5	2 (KM213458)	2 (KM213503)
38	M/3	ND	-	<5	3 (KM213459)	3 (KM213504)
39	F/14	2,3,4,6,9,10	*E. hartmanni*	<5	3 (KM213460)	3 (KM213505)
40	F/11	ND	*Cyclospora cayetanensis*	<5	3 (KM213461)	3 (KM213506)
41	F/14	2,3,9,10	-	<5	3 (KM213462)	3 (KM213507)
42	F/9	2,4,6,7	-	<5	3 (KM213463)	3 (KM213508)
43	M/6	2,3,4,8	*E. nana*	<5	3 (KM213464)	3 (KM213509])
44	F/6	2,5,10	*Trichomonas hominis*	>5	3 (KM213465)	3 (KM213510)
45	M/6	2,4,6,7,10	-	<5	3 (KM213466)	3 (KM213511)
46	M/2	2,6,7	-	<5	3 (KM213467)	3 (KM213512
47	M/8	2,4,7	-	<5	7 (KM213468)	7 (KM213513)

1, Bruxism; 2, Crams/Abdominal pain; 3, Bloating; 4, Constipation; 5, Vomiting; 6, Diarrhea; 7, Flatus; 8, Nausea; 9, Dizziness; 10, Headache; 11, Anorexia; *Parasite load, *Blastocystis* cells per 40X field; ^**‡**^SSUrDNA, small subunit rDNA gene; ^**§**^ITS, internal transcribed spacers (ITS-1 + 5.8S + ITS-2).

**Figure 1:**
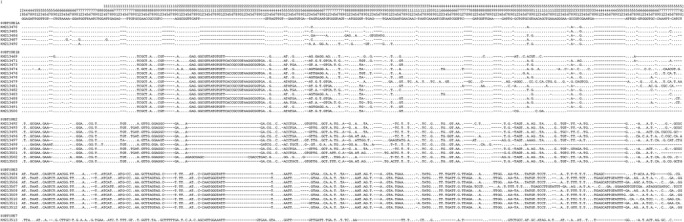
**Multiple sequence alignments.** Alignments of ITS1 + 5.8S + ITS2 of the rRNA gene of some representative *Blastocystis* STs (ST1, −2, −3 and −7) found in the present study.

**Figure 2:**
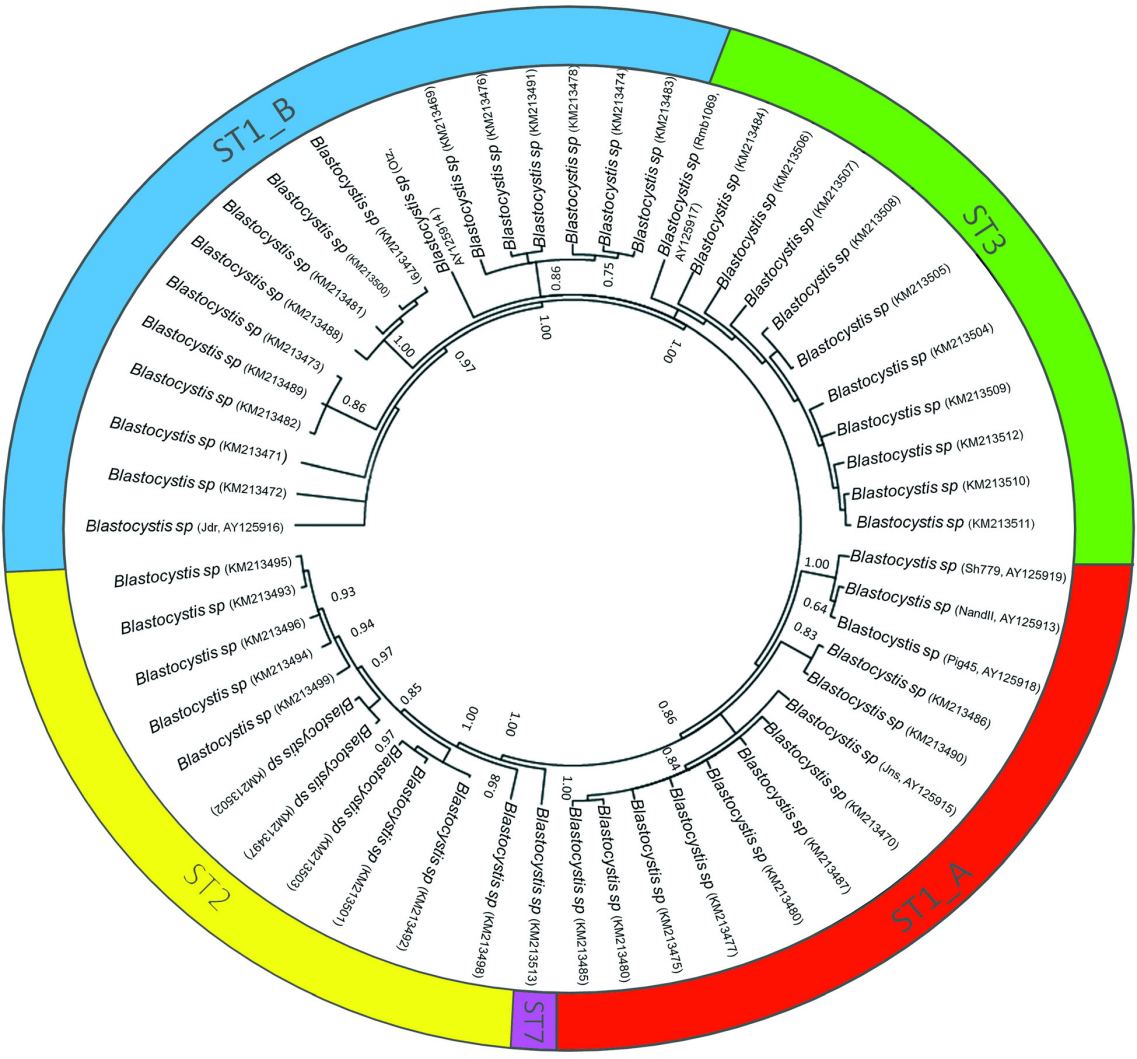
**Bayesian phylogenetic tree of** ***Blastocystis*** **STs from Mexico using ITS region sequences; the values of the nodes indicate posterior probabilities using 10 million generations.**

**Figure 3:**
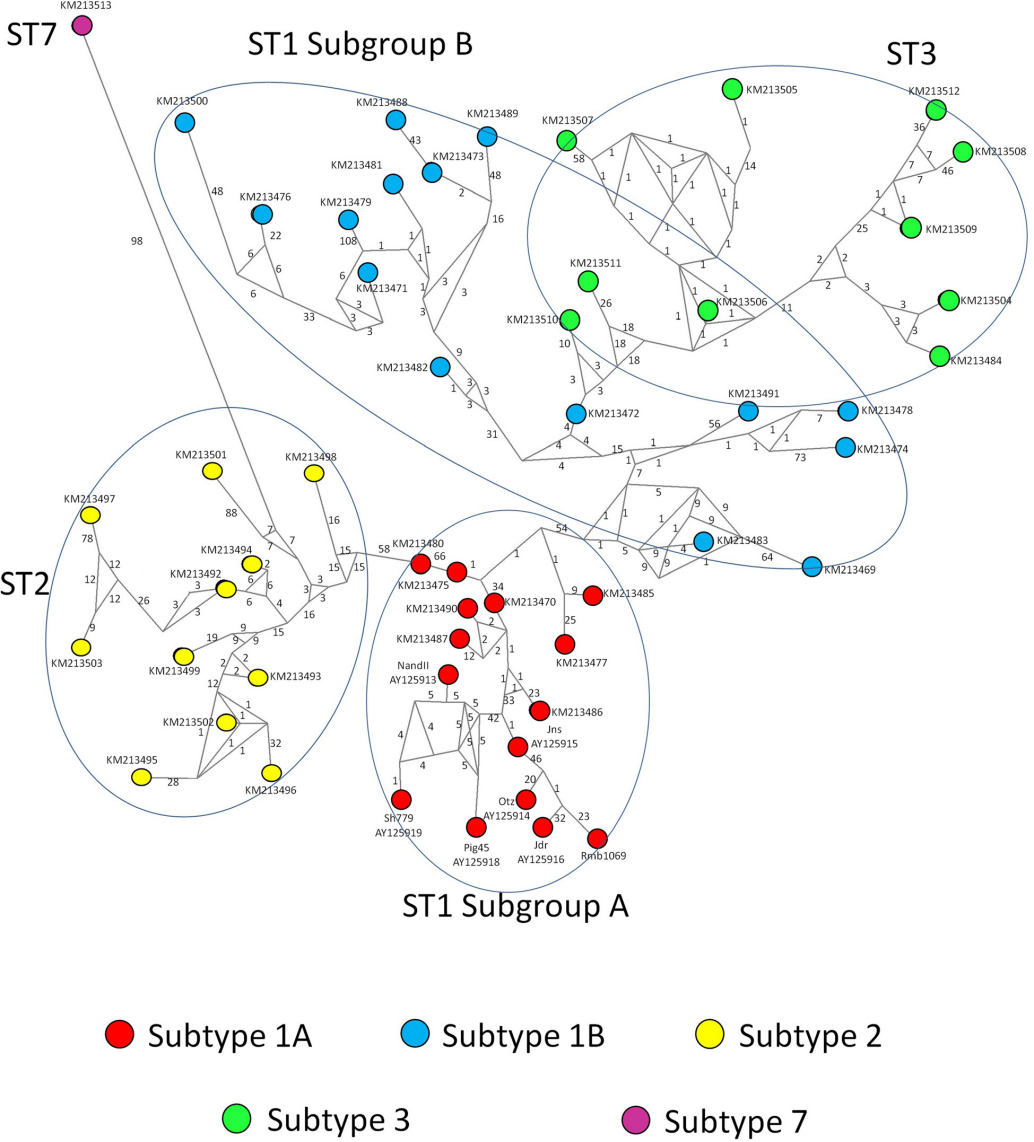
**Haplotype network tree using ITS region sequences of different** ***Blastocystis*** **STs; the numbers in branches refer to mutational changes.**

**Figure 4:**
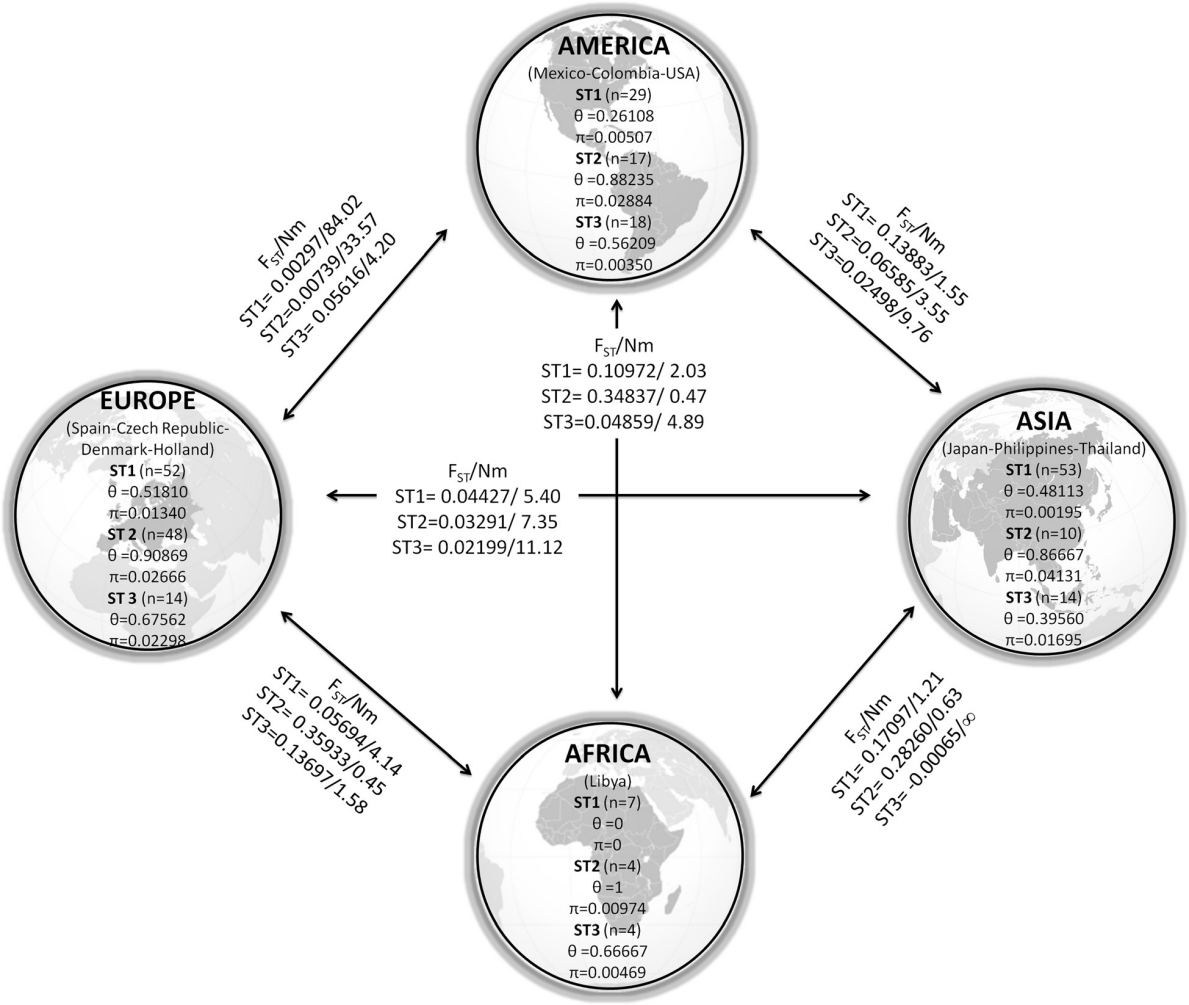
**Values of nucleotide diversity (π), haplotype polymorphism (θ), gene flow (Nm) and genetic differentiation index (F**_**ST**_ **) of** ***Blastocystis*** **STs by SSUrDNA analysis according to geographic area.**

Figure 1 shows selected alignments of the highly polymorphic ITS region used in this study, including representative sequences of each ST found. Clear nucleotide differences can be observed along the alignments within STs, particularly for ST1A and ST1B at nucleotide positions 181–242. Phylogenetic trees were built for the SSUrDNA (Figure not shown) and ITS (Figure 2) regions using all available sequences recorded in GenBank. Our ITS sequences were grouped into the clades ST1, −2, −3 and −7; interestingly, this inference also revealed the presence of two novel variants of ST1, clustering some sequences into ST1A and ST1B. The haplotype network (Figure 3) also exhibited the presence of two novel variants of ST1, which allowed for clearly distinguishing within each ST and showed the great genetic variability between them. The SSUrDNA (270) and ITS (56) sequences of *Blastocystis* ST1, −2 and −3 from Libya (Africa), Thailand-Philippines-Japan (Asia), Czech Republic-Denmark-Holland-Spain (Europe), and USA-Colombia (North and Latin America) deposited in the GenBank databases, as well as our sequences (accession numbers from KM213425 to KM213513), were analysed for their genetic variability. The average nucleotide diversity (π) and haplotype polymorphism (θ) for SSUrDNA was 0.0179 ± 0.0112 and 0.0355 ± 0.0076, respectively (Figure 4), with 0.0533 ± 0.0339 and 0.0642 ± 0.0428, respectively, found for ITS. The analysis regarding continents showed that π and θ were highest in the European population and lowest in the Libyan population (Figure 4, values inside of the continent image). Information on the π and θ data for human intestinal parasites is rather limited: for helminths, these values range from 0.004 to 0.025 for π and from 0.005 to 0.02 for θ [23-25]; for *Giardia lamblia*, π ranges from 0 to 0.07 [26]; and for *Entamoeba histolytica,* these values are π = 0.004 and θ = 0.51 (Martinez-Hernandez F, unpublished data).

The ratio coancestry coefficient (F_ST_)/migration index (Nm) for SSUrDNA showed the highest differentiation between the Libyan and Thailand-Philippines populations for ST2 (0.282/0.63); in contrast, a high flow gene was observed between the European and American populations for ST1 (0.003/84) (Figure 4, values over the arrows). As Africa had few sequences (ranged from 4 to 7), the differences in the genetic variation indexes were too large (ranging from 0 to 1).

In the present study, ITS was found to be more polymorphic than SSUrDNA (the ITS sequences showed 3 times more variants than SSUrDNA) and thus is a good marker for research on genetic diversity, as for other parasites [9,10,24,27,28]. Therefore, its use in population genetic analyses offers reliable results on the genetic variability within populations of this parasite. Regardless, as there are only a few sequences of *Blastocystis* ITS regions available in GenBank, a robust population genetic analysis using this marker is not possible. In addition, because ITS allows for distinguishing between ST1, −2, −3 and −7 using only one PCR directly applied to faecal samples, this marker could be used for *Blastocystis* ST diagnosis, avoiding the use of PCR-sequencing [11]. However, a limiting factor for this genetic marker is the lack of information about the length of the ITS region of other relevant human STs, such as ST4. An interesting finding in the present study was that the ITS analysis allowed for the clear identification of two novel variants of ST1, as recognised by Bayesian and haplotype network inferences. Recently, Ramírez *et al*. [29] analysed several faecal samples containing *Blastocystis* recovered from humans, birds and other mammals; the ST identification was performed according to DNA barcoding, and a phylogenetic reconstruction using a Maximum Composite Likelihood was performed. Interestingly, the ST1 cluster exhibited two clades, indicating variants, but this was not sufficiently discussed.

The SSUrDNA analysis revealed that ST1 and ST2 showed the lowest genetic differentiation, with a high gene flow between the American and European populations, whereas the match between ST1 and ST2 for the Libyan and American populations showed a higher genetic differentiation with little gene flow. Interestingly, ST3 exhibited the most gene flow (migrants) for Thailand-Philippines vs Libya, America and Europe. Libya had a low number of sequences (ranging from 4 to 7); thus, differences in the variation of the genetic values were also large (ranging from 0 to 1). Alfellani *et al.* [2] determined the prevalence of *Blastocystis* STs in populations from Africa and UK and compiled and contrasted the relative distribution of *Blastocystis* STs across continents, finding a significant variation in ST prevalence between populations. Our results support the analysis of Alfellani *et al*. [2] because for ST3, these authors found frequencies up to 40% for major geographic regions, except for America, where the frequency was approximately 25%. According to our F_ST_/Nm data, the gene flow (Nm) of ≈ 4 for America-Europe and America-Africa as well as for America-Asia (Nm ≈ 9) suggest a high migrant flow, but less than Europe-Asia (Nm ≈ 12) and Africa-Asia (Nm ≈ ∞), indicating a vast migrant flow among these areas. For ST2, America shows the highest frequency (up to 30%), with low values of Nm <4 for America-Africa and America-Asia but Nm = 33 for America-Europe; these results could mean that ST2 from America is more isolated than in other major geographic regions. Finally, ST1 shows contrasting values because it was more frequent in America and East/Southeast Asia and lower in Europe and Africa. The F_ST_/Nm data showed Nm ≤ 4 among Africa vs Europe-America-Asia, whereas Nm ranged from 4 to 84 between Europe and America-Asia-Africa. Therefore, due to the current globalisation, the variable frequencies observed in ST prevalence might indirectly show the genetic differentiation and gene flow of each ST population around the world.

Poirier *et al*. [30] described high polymorphism between copies of the SSUrDNA gene of *Blastocystis* ST7 and Stensvold *et al.* [7,31] emphasised a remarkable intra-ST genetic diversity and some cryptic host specificity for *Blastocystis*. Considering that we used part of SSUrDNA in our genetic analyses, we can speculate that these biological features may be a secondary effect due to variation in other genes; most likely by a direct genetic hitchhiking phenomenon [32] or by an indirect association, similar to what has been proposed for other parasites [33].

## Abbreviations

ITS: Internal transcribed spacers
STs: Subtypes
SSUrDNA: Small subunit rDNA gene
π: Nucleotide diversity
θ: Haplotype polymorphism
F_ST_: Genetic differentiation index
Nm: Migration index
